# Baseline HIV Genotyping and Antiretroviral Therapy Resistance Mutations in Saudi Arabian Population, a Multicentre, Cross-Sectional Study

**DOI:** 10.3390/v18080820

**Published:** 2026-07-26

**Authors:** Roa Al-Osaimi, Moayad Al-Qurashi, Lama Al-Zamil, Batool Ali, Reem Al-Mutairy, Ali Al-Saeed, Meqbel Al-Shelawi, Abdullah Al-Khalaf, Abdullah Al-Subaie, Layla Faqih

**Affiliations:** 1 Department of Adult Infectious Diseases, Jeddah First Health Cluster, East Jeddah General Hospital, Jeddah 22253, Saudi Arabia; 2 Division of Adult Infectious Diseases, Department of Medicine, King Fahad Armed Forces Hospital, Ministry of Defence—Health Services, Jeddah 23311, Saudi Arabia; 3 Department of Clinical Laboratories Sciences, College of Applied Medical Sciences, King Saud University, Riyadh 11433, Saudi Arabia; 4 Pharmaceutical Care Department, King Fahad Armed Forces Hospital, Ministry of Defence—Health Services, Jeddah 23311, Saudi Arabia; 5 Department of Infectious Diseases, Dammam Medical Complex of the Eastern Health Cluster, Dammam 32245, Saudi Arabia; 6 Department of Medicine, Jeddah First Health Cluster, East Jeddah General Hospital, Jeddah 22253, Saudi Arabia; 7 Department of Medicine, Dammam Medical Complex of the Eastern Health Cluster, Dammam 32245, Saudi Arabia

**Keywords:** HIV, epidemiology, genotyping, Saudi Arabia

## Abstract

Background: Transmitted drug resistance (TDR) remains a critical challenge in HIV-1 management, particularly in treatment-naïve populations. Baseline genotypic resistance testing is recommended to optimize antiretroviral therapy (ART), yet data from Saudi Arabia remain limited. This study aimed to characterize HIV-1 genetic diversity and baseline resistance-associated mutations among newly diagnosed, ART-naïve individuals. Methods: We conducted a multi-centre, retrospective cross-sectional study across three hospitals in Saudi Arabia between January 2023 and December 2024. Adult, treatment-naïve patients with confirmed HIV infection who underwent genotyping using Sanger sequencing were included. Mutations in reverse transcriptase (RT), protease (PI), and integrase (INSTI) genes were analyzed using the Stanford HIV Drug Resistance Database. Demographic, clinical, and virological data were collected, and comparative analyses between regions were performed. Results: A total of 614 patients were included, predominantly male (85.5%) and aged 25–44 years. Most patients presented with high viral loads (≥10,000 copies/mL, 89.7%), and 25.3% had CD4 counts <200 cells/mm^3^. HIV-1 subtype distribution was highly diverse, with subtype C (19.5%), CRF02_AG (15.6%), and subtype G (12.7%) predominating. Although mutations were frequently detected (RT: 85.2%, PI: 94.7%, INSTI: 36.4%), the majority were subtype-associated polymorphisms rather than major drug resistance mutations. Clinically significant mutations including M184V/I (1.17%), K103N (0.83%), and K65R (0.17%) were observed at low frequencies. No major INSTI resistance mutations were detected. Multi-class mutation patterns were common but largely driven by accessory variants. Conclusions: Despite the high prevalence of detected mutations, clinically significant TDR remained low, occurring in approximately 2.8% of patients. Most detected variants were polymorphic or accessory mutations, while susceptibility to INSTIs remained largely preserved. Continued baseline genotyping and molecular surveillance remain important to monitor emerging resistance patterns.

## 1. Introduction

Despite remarkable progress in antiretroviral therapy (ART), HIV-1 remains a global public health challenge due to its high genetic diversity and the persistence of drug-resistant mutations [1]. A major concern is the emergence and transmission of drug-resistant strains, even among individuals who have never received treatment. This phenomenon, known as transmitted drug resistance (TDR), can undermine the effectiveness of first-line regimens and compromise long-term treatment outcomes [2,3].

Baseline genotypic resistance testing has therefore become an important tool in HIV care. By identifying TDR prior to therapy initiation, clinicians can select effective first-line regimens, reduce the risk of virologic failure, and improve durability of ART. Beyond individual benefits, baseline testing contributes to public health surveillance by monitoring resistance trends and informing guideline development [2,3,4]. Although a systematic review found no direct evidence linking baseline testing to improved clinical outcomes [5], cost-effectiveness analyses demonstrated its value in settings where NNRTI resistance exceeds 1.5% [6].

International guidelines vary in their recommendations. The U.S. Department of Health and Human Services (DHHS) and the International AIDS Society–USA (IAS–USA) endorse baseline genotyping at HIV diagnosis, while the British HIV Association (BHIVA) similarly recommends resistance testing for all newly diagnosed patients, regardless of CD4 count or viral load. Historically, such testing was most critical when NNRTIs and protease inhibitors (PIs) were the foundation of ART. More recently, the widespread adoption of second-generation integrase strand transfer inhibitors (INSTIs), which have high genetic barriers to resistance, has led some guidelines to move away from universal baseline testing. Nevertheless, in regions where TDR prevalence is high, particularly for NNRTIs, baseline testing remains essential, and WHO now recommends INSTI-based first-line therapy [7,8,9].

Surveillance studies underscore these concerns. In the United States, Hugueley et al. (2022) found that although baseline resistance testing uptake was suboptimal, integrase resistance testing increased to 23.5% by 2019, supporting its growing role in outbreak detection and resistance monitoring [10]. Globally, NNRTI resistance has reached concerning levels, exceeding 10–20% in some low- and middle-income countries, while NRTI and PI resistance remain lower. Encouragingly, major INSTI resistance mutations remain rare in treatment-naïve populations [11,12,13].

In Saudi Arabia, however, data on TDR are limited. Available studies are few, often single-center, and involve small cohorts. Reported resistance rates among treatment-naïve patients include approximately 7% for PIs, 8.8% for NRTIs, and 5.4% for NNRTIs [14]. These findings suggest that resistance mutations are present locally, though the true prevalence remains uncertain. Research into dormant resistance-associated mutations is particularly scarce, despite their potential to compromise ART if undetected before therapy initiation.

This study therefore aimed to evaluate dormant resistance-associated mutations in the HIV-1 genome among newly diagnosed, treatment-naïve individuals in Saudi Arabia, in order to identify circulating resistant strains, inform first-line treatment strategies, and support future national policy on baseline resistance testing.

## 2. Materials and Methods

### 2.1. Study Cohort

This is a multi-centre, retrospective cross-sectional study conducted at three hospitals from Saudi Arabia: East Jeddah General Hospital and King Fahad Armed Forces Hospital from Jeddah at the western region and Dammam Medical Complex from the eastern region. Spanning from January 2023 to December 2024. The study targets adult HIV-positive patients from both inpatient and outpatient settings who are antiretroviral treatment (ART)-naïve. Participants were eligible for inclusion if they were adults aged ≥18 years with a confirmed diagnosis of HIV, were ART-naïve at the time of genotyping, and had HIV genotyping performed using the Sanger sequencing method. Individuals were excluded if they were younger than 18 years, had an unconfirmed HIV diagnosis (e.g., lacking confirmation by Western blot or PCR), had initiated ART or received pre- or post-exposure prophylaxis, or if genotyping was conducted using methods other than Sanger sequencing. Additional exclusion criteria included incomplete clinical or laboratory data that could compromise study integrity, as well as individuals classified as elite controllers.

### 2.2. Data Collection

Patients’ demographics, comorbidities, along with the outcomes of antiretroviral therapy, were extracted from the electronic medical records. This information was sourced from the database of the pathology and clinical laboratory medicine in all hospitals. All data related to patients’ personal information, previous interventions for HIV adhered strictly to the stipulated data protection guidelines.

Data were collected electronically using a structured Excel sheet. Variables collected include date of birth, age, gender, date of HIV diagnosis, clinical outcome, current ART regimen (if applicable), and identified genetic mutations.

### 2.3. Genotypic and Mutation Analysis

HIV-1 genotypic resistance data were obtained retrospectively from the original clinical genotypic resistance reports generated during routine diagnostic care. As this was a retrospective study, genotypic testing had already been performed by two accredited external reference laboratories as part of standard clinical management rather than specifically for this study. Samples from the participating hospitals were analyzed by either Hôpitaux Universitaires de Genève (HUG), Geneva, Switzerland, or Al Borg Diagnostics, Saudi Arabia, according to the referral pathway in place at the time of testing.

Genotypic resistance testing was performed using Sanger sequencing of HIV-1 RNA extracted from EDTA plasma. Patient sequence analyses targeting the RT, PI, and INSTI regions. The standard clinical specimen for HIV drug resistance testing, representing circulating virus. Identified mutations were classified according to the Stanford HIV Drug Resistance Database and grouped by drug class. Mutations were categorized as pure subtype, recombinant (CRF/URF), unclassified, or unreported. The frequency and co-occurrence of mutations were summarized. Mutations with a prevalence ≥5% were considered frequent, while clinically relevant major mutations occurring at <5% were separately reported. Multi-class resistance patterns were evaluated by combining the presence of mutations across RT, PI, and INSTI genes.

HIV-1 subtype information was extracted from the original clinical genotypic resistance reports generated during routine diagnostic testing. Subtype assignments, when available, were recorded as reported by the performing laboratory and categorized as pure subtype, circulating recombinant form (CRF), unique recombinant form (URF), or unclassified. No independent subtype re-analysis or phylogenetic reconstruction was performed for this study.

### 2.4. Ethical Consideration

All data were stored on password-protected computers accessible only to authorized investigators at each participating center. Patient data were anonymized and coded to ensure confidentiality and allow secure sharing between centers without disclosing personal identifiers.

Ethical clearance for the study was granted by the Institutional Review Board of each centre separately with the following reference numbers (REC 678, A01861 and IM-100) in accordance with the Declaration of Helsinki and the relevant guidelines and regulations. Informed consent was waived due to the retrospective nature of the study.

### 2.5. Statistical Analysis

All analyses were conducted using Microsoft Excel (Microsoft Corp., Redmond, WA, USA) and GraphPad Prism 11.0.0 (GraphPad Software, San Diego, CA, USA). Descriptive statistics were used to summarize categorical and continuous variables. Frequencies and percentages were computed for demographic and clinical variables. The relative risk (RR) with corresponding 95% confidence intervals (CIs) was calculated to compare characteristics between cities (Jeddah vs. Dammam). A *p*-value < 0.05 was considered statistically significant. Mutation frequencies were calculated using the number of successfully genotyped sequences available for each gene region rather than the total study population. Gene-specific denominators were 600 for reverse transcriptase (RT), 603 for protease (PI), and 574 for integrase (INSTI). In contrast, analyses of multi-class mutation profiles were performed using the entire study cohort (*n* = 614), with patients classified according to the presence or absence of mutations across the available gene regions.

## 3. Results

### 3.1. Demographic, Clinical Characteristics and Comparative Analysis by City

A total of 614 participants were included in the study (Table 1). The majority were aged 25–34 years (41.2%), followed by 35–44 years (28.8%), with a mean age skewed toward young and middle adulthood. Most patients were male (85.5%) and resided in Jeddah (72.3%), while the remainder were from Dammam (27.7%). At diagnosis, the largest proportion of patients were between 25 and 34 years (44%). Clinical outcomes indicated that 87.9% of patients were virologically controlled, while 4.2% were uncontrolled, 4.9% were lost to follow-up, and 2.9% had died. Immunological status varied, with 25.3% presenting with CD4 counts <200 cells/mm^3^ and 33.4% with CD4 ≥ 500. Viral load data showed that the majority (89.7%) had ≥10,000 copies/mL at baseline. With regard to viral clades, 43.5% were pure subtypes, 34.9% were recombinant (CRF/URF), 0.3% were unclassified and 21.3% were not done/mentioned in the genotyping report.

Relative risk analysis was performed to compare demographic, clinical, and virological characteristics of patients from Jeddah (*n* = 444) and Dammam (*n* = 170) (Table 1). Gender distribution did not differ significantly between cities, with males comprising the majority in both cohorts (85.4% vs. 85.9%, RR = 0.99, 95% CI: 0.93–1.07, *p* = 1.0). Age distribution revealed significant differences. Patients aged 25–34 years were more common in Dammam (50.0%) than Jeddah (37.8%), corresponding to a lower relative risk in Jeddah (RR = 0.76, 95% CI: 0.62–0.92, *p* = 0.007). Conversely, patients aged 45–54 years were significantly more prevalent in Jeddah (14.0%) compared to Dammam (7.1%) (RR = 1.98, 95% CI: 1.09–3.58, *p* = 0.018). Trends toward higher proportions of patients aged 55–64 years were also observed in Jeddah, though this did not reach statistical significance (*p* = 0.068). Age at diagnosis showed a similar pattern, with patients aged 45–54 years more frequently diagnosed in Jeddah than Dammam (14.0% vs. 7.7%, RR = 1.83, 95% CI: 1.03–3.23, *p* = 0.038). Other age groups did not differ significantly between the two cities. Clinical outcomes were strikingly different. All patients in Dammam were reported as clinically controlled, whereas in Jeddah, 83.3% were controlled, with small proportions reported as missed follow-up (6.8%), uncontrolled (5.9%), or deceased (4.1%). This translated into a significantly lower likelihood of virologic control in Jeddah (RR = 0.83, 95% CI: 0.80–0.87, *p* < 0.0001). Immunological status (CD4 count) also differed significantly. Patients in Jeddah were more likely to present with advanced immunosuppression (CD4 < 200 cells/mm^3^: 27.7% vs. 11.8%, RR = 2.35, 95% CI: 1.52–3.65, *p* < 0.0001). In contrast, high CD4 counts (≥500 cells/mm^3^) were more frequent in Dammam (55.3% vs. 21.4%), with Jeddah patients showing a markedly lower likelihood of this outcome (RR = 0.39, 95% CI: 0.31–0.48, *p* < 0.0001). Viral load patterns were consistent with the above. Nearly all patients in Dammam had high viral loads (≥10,000 copies/mL: 100% vs. 80.9%), whereas smaller subsets of Jeddah patients fell into lower viral load categories (1000–9999: 11.3%, <1000: 2.5%). Patients from Jeddah were therefore significantly less likely to present with very high viral loads compared to Dammam (RR = 0.81, 95% CI: 0.77–0.84, *p* < 0.0001). Clade distribution showed highly significant differences. Pure subtypes were more common in Jeddah (53.4% vs. 17.7%, RR = 3.02, 95% CI: 2.16–4.23, *p* < 0.0001), as were recombinant forms (41.9% vs. 16.5%, RR = 2.54, 95% CI: 1.78–3.63, *p* < 0.0001). Conversely, “not mentioned” clades were overwhelmingly more frequent in Dammam (65.9% vs. 4.3%, RR = 0.06, 95% CI: 0.04–0.10, *p* < 0.0001), reflecting reporting or testing inconsistencies between centres.

### 3.2. Distribution of HIV-1 Clades/Subtypes

**Figure 1:**
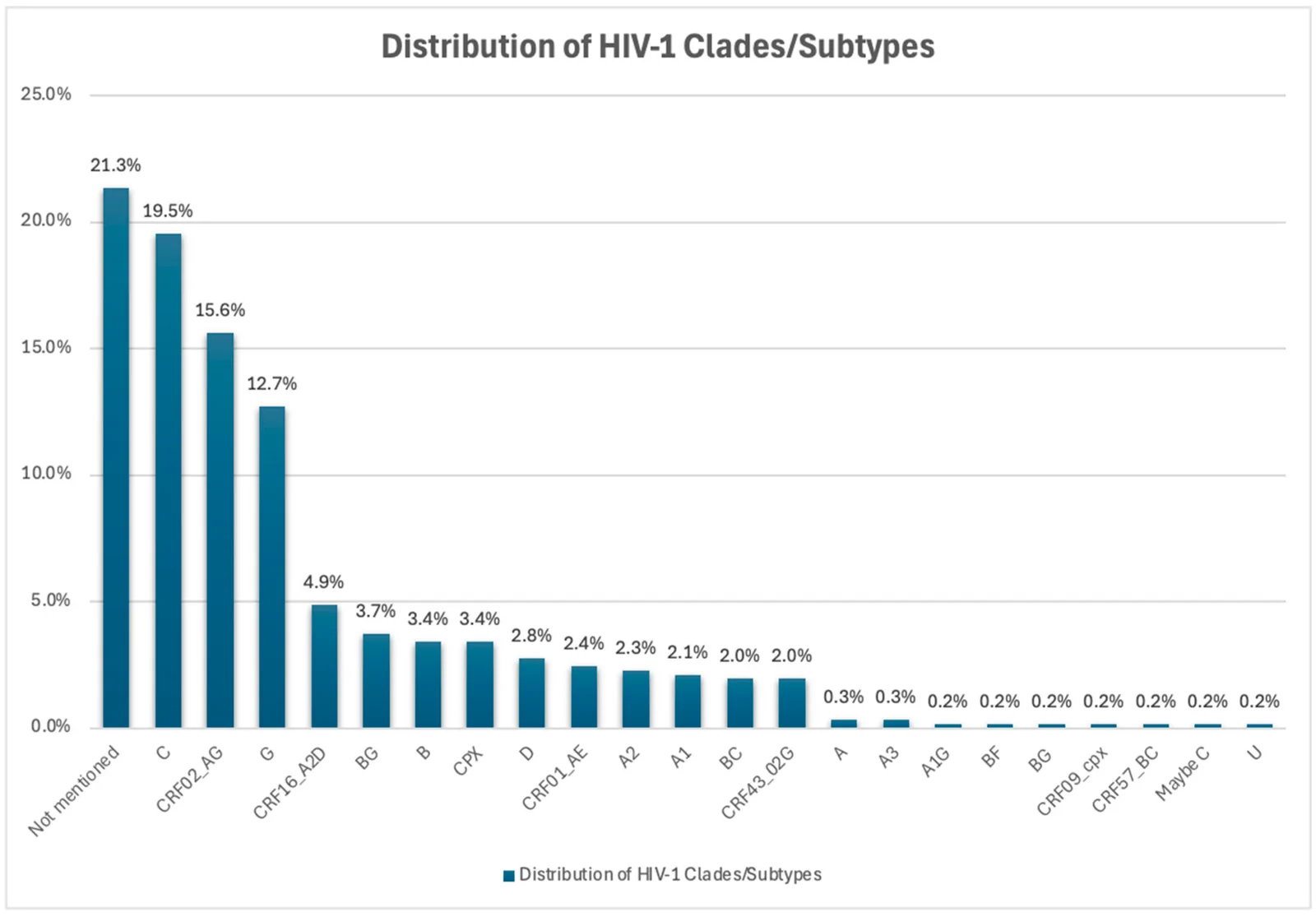
Distribution of HIV-1 clades and subtypes in the study cohort. Distribution of HIV-1 clades and subtypes among treatment-naïve individuals in the study cohort (*n* = 614). Subtype classification revealed a predominance of non-B variants, with subtype C (19.5%), CRF02_AG (15.6%), and subtype G (12.7%) representing the most common identified clades. A substantial proportion of cases (21.3%) were classified as “not mentioned,” reflecting incomplete subtype reporting.

The distribution of HIV-1 clades and subtypes in the cohort demonstrated substantial genetic diversity (Figure 1). A notable proportion of cases (21.3%, *n* = 131) were classified as “not mentioned,” indicating incomplete subtype reporting. Among identified subtypes, subtype C was the most prevalent (19.5%, *n* = 120), followed by CRF02_AG (15.6%, *n* = 96) and subtype G (12.7%, *n* = 78). Other circulating recombinant forms and subtypes were present at lower frequencies, including CRF16_A2D (4.9%), BG (3.7%), subtype B (3.4%), and CPX (3.4%). Less common variants included subtype D (2.8%), CRF01_AE (2.4%), A2 (2.3%), A1 (2.1%), BC (2.0%), and CRF43_02G (2.0%). Rare subtypes and recombinants—such as A, A3, A1G, BF, CRF09_cpx, CRF57_BC, “Maybe C,” and unclassified (U)—were each observed in ≤0.3% of the cohort. Overall, the findings highlight a predominance of non-B subtypes and recombinant forms, reflecting a highly heterogeneous HIV epidemic within the study population.

### 3.3. Prevalence of Drug Resistance Mutations

Although mutations were frequently detected across all antiretroviral drug classes (Table 2), the majority represent polymorphic or accessory substitutions rather than major drug resistance mutations (DRMs). Reverse transcriptase (RT)-associated mutations were identified in 85.2% of patients; however, the most common variant, R211K (59.5%), along with S68G, D177E, and A98S, are generally considered polymorphic or accessory mutations with limited impact on drug susceptibility when present in isolation.

Similarly, protease inhibitor (PI)-associated mutations were observed in 94.7% of patients and were dominated by well-characterized subtype-associated polymorphisms such as M36I, H69K, L89M, I13V, and K20I. These mutations are highly prevalent in non-B HIV-1 subtypes and are not classified as major surveillance drug resistance mutations (SDRMs), although they may contribute to resistance pathways when combined with major mutations under selective pressure.

Integrase strand transfer inhibitor (INSTI)-associated mutations were less common (36.4%) and consisted primarily of accessory variants, including M50I, L74I, and G163E, which have minimal clinical impact in the absence of major INSTI resistance mutations. Importantly, no major INSTI resistance mutations—such as Q148H/K/R, N155H, or Y143C/H/R—were detected, indicating preserved susceptibility to second-generation INSTIs.

### 3.4. Multi-Class Resistance Profiles

As shown in Table 3, the majority of patients harbored multi-class resistance mutations. RT + PI dual-class mutations were most common (53.4%), followed by triple-class mutations (RT + PI + INSTI) in 29.5%. PI-only resistance was observed in 6.7%, while INSTI-only (1.3%) and RT-only (0.3%) profiles were rare. Only 5.4% of patients had no detectable resistance mutations.

### 3.5. Frequency of Individual Mutations and Clinical Relevance

As show in Table 4, the mutation profile of this cohort was characterized by a high prevalence of substitutions; however, the majority were polymorphic or accessory mutations rather than clinically significant drug resistance mutations. Among reverse transcriptase (RT) mutations occurring at ≥5% frequency, R211K was the most common (59.5%), followed by S68G, D177E, A98S, and Q207E. These variants are predominantly considered natural polymorphisms or accessory mutations with minimal impact on antiretroviral susceptibility when present alone. Notably, A98S represents a minor NNRTI-associated mutation that may contribute to resistance only when combined with major NNRTI mutations.

Protease inhibitor (PI)-associated mutations were highly prevalent, with M36I (84.1%), H69K (78.9%), and L89M (75.6%) observed in the majority of patients. These mutations are well-established subtype-associated polymorphisms commonly found in non-B HIV-1 strains and are not classified as major resistance mutations. Additional variants, including I13V, K20I, L10I, and T74P, further reflect a background of accessory mutations that may facilitate resistance development under drug pressure but do not independently confer clinically meaningful resistance.

In the integrase (INSTI) region, mutations were less frequent and consisted primarily of accessory substitutions such as M50I, L74I, and G163E. These mutations are known to have minimal clinical impact in isolation and typically require the presence of major resistance mutations to significantly reduce susceptibility to integrase inhibitors.

Importantly, clinically significant major drug resistance mutations were identified at low frequencies (<5%). These included M184V/I (1.17%), a key mutation conferring high-level resistance to lamivudine and emtricitabine; K65R (0.17%), associated with reduced susceptibility to tenofovir and abacavir; and NNRTI mutations such as K103N (0.83%) and Y181C (0.17%), which are strongly linked to efavirenz and nevirapine resistance. Major INSTI resistance mutations, including Q148H/K/R (0.35%) and N155H (0.17%), were rare.

### 3.6. Co-Occurrence of Mutations

Analysis of co-occurrence patterns (Table 5) showed frequent clustering of PI polymorphisms, with M36I + H69K (73.1%), M36I + L89M (69.8%), and H69K + L89M (69.5%) being the most common combinations. In RT genes, R211K frequently co-occurred with A98S (5.2%), Q207E (3.5%), and D177E (3.2%). Co-occurrence of INSTI mutations were rare, with G163E + L74I (1.4%) being the most frequent combination.

## 4. Discussion

Baseline genotypic resistance testing remains a cornerstone in the management of newly diagnosed HIV patients. Its primary purpose is to identify transmitted drug resistance (TDR), which, if left undetected, can compromise first-line regimens, precipitate early virologic failure, and restrict future treatment options. By detecting resistance mutations before therapy initiation, clinicians can avoid ineffective regimens and optimize long-term outcomes. Globally, baseline resistance testing has demonstrated both clinical and economic value: while associated with upfront costs, it prevents treatment failure, reduces the need for costly second-line regimens, and is considered cost-effective in settings where NNRTI resistance exceeds 1.5%. Importantly, baseline genotyping also informs public health by monitoring resistance trends, identifying transmission clusters, and guiding evidence-based treatment guidelines [6,11,12,13,14,15].

A particular concern is the presence of dormant mutations, resistance-associated variants that exist before treatment initiation, either as natural polymorphisms or through transmission. These mutations, particularly within the NRTI and NNRTI classes, have been linked to early treatment failure when not identified [16]. Their presence reinforces the need for baseline resistance testing, even in settings where high-barrier INSTI-based regimens are widely adopted [9,11].

In Saudi Arabia, information on HIV drug resistance remains limited and is mainly derived from small, single-centre studies. One study reported that among treatment-naïve patients, resistance prevalence was highest for NRTIs at 8.8%, followed by protease inhibitors at 7%, and NNRTIs at 5.4% [14]. While these findings confirm the circulation of resistant strains, they may not reflect the broader epidemic due to limited scope and inconsistent genotyping practices. The lack of nationwide surveillance and integration of resistance testing into routine care represents a significant gap in local HIV management. Addressing this will require large, multi-centre studies with standardized genotyping, expanded molecular surveillance, and national-level investments in laboratory capacity, clinician awareness, and policy guidance.

This multi-centre study provides one of the most comprehensive assessments of HIV-1 genetic diversity and baseline resistance-associated mutations among treatment-naïve individuals in Saudi Arabia. The findings highlight a highly heterogeneous epidemic characterized by a predominance of non-B subtypes, widespread polymorphic mutations, and a comparatively low prevalence of clinically significant TDR.

A key observation in this study is the apparent high frequency of mutations across all drug classes; however, careful interpretation reveals that the majority represent subtype-associated polymorphisms or accessory mutations rather than major DRMs. For example, the most prevalent protease mutations (M36I, H69K, L89M, I13V) and reverse transcriptase substitutions (R211K, D177E, Q207E) are well-documented natural variants in non-B subtypes. While these mutations were detected in a large proportion of patients, they have minimal impact on antiretroviral susceptibility when present in isolation. This distinction is critical, as overinterpretation of polymorphic mutations may lead to an overestimation of resistance burden at the population level.

In contrast, true clinically relevant DRMs—such as M184V/I, K65R, K103N, and Y181C—were identified at low frequencies (<2% for most mutations). These findings suggest that, despite the widespread presence of mutations, the actual prevalence of transmitted drug resistance in this cohort remains relatively low. This aligns with previous reports from Saudi Arabia and contrasts with higher resistance rates observed in some low- and middle-income countries. Importantly, the low prevalence of major INSTI resistance mutations (e.g., Q148H/K/R, N155H) supports the continued effectiveness of second-generation integrase inhibitors such as dolutegravir and bictegravir as first-line therapy.

The extensive co-occurrence of polymorphic mutations, particularly within the protease gene, further reflects the underlying subtype distribution rather than treatment-driven resistance. Frequent clustering of mutations such as M36I, H69K, and L89M is characteristic of non-B subtypes and may represent evolutionary adaptation rather than selective drug pressure. Nonetheless, these accessory mutations may lower the genetic barrier to resistance and facilitate the emergence of major mutations under antiretroviral exposure, underscoring the importance of ongoing surveillance.

The clade distribution observed in this study reinforces the genetic complexity of HIV-1 in Saudi Arabia [17,18]. The predominance of subtype C, CRF02_AG, and subtype G, along with a substantial proportion of recombinant forms, suggests a diverse epidemic influenced by multiple transmission networks. While historically such diversity has been associated with travel and migration, the consistent presence of similar subtype patterns and mutation profiles across patients raises the possibility of increasing local transmission of HIV, including strains carrying resistance-associated variants. This has important public health implications, as it indicates that resistant viruses may be circulating within local communities rather than being solely imported.

From a clinical perspective, the findings support the continued use of INSTI-based regimens as the preferred first-line therapy, given the absence of major integrase resistance mutations. However, the detection of low-frequency but clinically significant NRTI and NNRTI mutations highlights the importance of baseline genotypic resistance testing, particularly in optimizing individualized treatment strategies and preventing early virologic failure. Overall, clinically significant TDR, defined by the presence of at least one major surveillance drug resistance mutation (SDRM), was identified in approximately 2.8% of patients. Additionally, the predominance of HIV-1 subtype C highlights the importance of strict adherence to antiretroviral therapy, as this subtype/clade is associated with a higher propensity for the selection of the K65R resistance mutation under tenofovir pressure. This phenomenon is attributed to distinct reverse transcriptase template sequence motifs in subtype C that facilitate more rapid emergence of K65R compared with other subtypes [19,20,21].

Another important consideration is the lack of assessment of APOBEC-mediated hypermutation in this study. Hypermutation is increasingly recognized as a contributor to apparent resistance mutations, particularly G-to-A substitutions, which may not reflect replication-competent virus. Failure to identify and exclude hypermutated sequences can lead to misinterpretation of resistance profiles and overestimation of clinically relevant mutations [22]. Incorporating hypermutation screening in future studies would enhance the accuracy of resistance analysis and provide deeper insights into viral evolution.

Geographic differences between Jeddah and Dammam further highlight disparities in clinical presentation and outcomes. Patients in Jeddah were more likely to present with advanced immunosuppression and lower rates of virologic control, whereas patients in Dammam demonstrated better immunological status but higher baseline viral loads. These differences may reflect variations in healthcare access, diagnostic practices, or timing of presentation, and emphasize the need for standardized care pathways across regions.

This study has several limitations. Its retrospective cross-sectional design limits the ability to assess temporal trends or causal relationships. The use of Sanger sequencing may underestimate minority resistance variants, the lack of INSTI genotyping in some reports that underestimates the true prevalence of INSTI DRMs and the absence of hypermutation analysis may affect mutation interpretation. Additionally, incomplete subtype reporting in a subset of patients may limit the accuracy of epidemiological conclusions. Because subtype assignments were obtained from routine clinical reports rather than reanalyzed using a standardized subtyping algorithm, methodological differences between laboratories may have influenced subtype classification.

In conclusion, this study demonstrates that while mutations are highly prevalent among treatment-naïve individuals in Saudi Arabia, the majority are polymorphic and subtype-related rather than indicative of true drug resistance. The low prevalence of major DRMs and preserved susceptibility to INSTIs are encouraging for current treatment strategies. However, the presence of diverse subtypes, emerging local transmission patterns, and low-frequency resistance mutations underscores the need for continued molecular surveillance, standardized genotyping practices, and consideration of baseline resistance testing to optimize HIV care and inform national treatment policies.

## Figures and Tables

**Table 1:** Demography of participants (*n* = 614) and Relative Risk (RR) of Characteristics by City (Jeddah vs. Dammam).

Variable	Category	N	*%*	Risk in Jeddah (%)	Risk in Dammam (%)	RR	95% CI	*p*-Value	
Gender	Male	525	85.5	85.36	85.88	0.99	(0.93, 1.07)	1	
	Female	89	14.5	14.64	14.12	1.04	(0.67, 1.60)	1	
Age	15–24	41	6.7	5.86	8.82	0.66	(0.36, 1.22)	0.206572805	
	25–34	253	41.2	37.84	50.00	0.76	(0.62, 0.92)	0.007783811	**
	35–44	177	28.8	29.95	25.88	1.16	(0.86, 1.55)	0.370251038	
	45–54	74	12.1	13.96	7.06	1.98	(1.09, 3.58)	0.0184244	*
	55–64	50	8.1	9.46	4.71	2.01	(0.96, 4.19)	0.068447033	
	65+	19	3.1	2.93	3.53	0.83	(0.32, 2.15)	0.794697025	
Age of Diagnosis	<15	1	0.2	0.23	0.00	-	-	-	
	15–24	65	10.6	9.46	13.53	0.70	(0.43, 1.13)	0.145113719	
	25–34	270	44	42.12	48.82	0.86	(0.71, 1.04)	0.146318309	
	35–44	158	25.7	26.80	22.94	1.17	(0.85, 1.60)	0.354263382	
	45–54	75	12.2	13.96	7.65	1.83	(1.03, 3.23)	0.038161742	*
	55–64	34	5.5	5.63	5.29	1.06	(0.51, 2.23)	1	
	65+	11	1.8	1.80	1.76	1.02	(0.27, 3.80)	1	
Clinical Status	Controlled	540	87.9	83.33	100.00	0.83	(0.80, 0.87)	1.12 × 10^−11^	****
	Missed	26	4.2	6.76	0.00	-	-	-	
	Deceased	30	4.9	4.05	0.00	-	-	-	
	Uncontrolled	18	2.9	5.86	0.00	-	-	-	
CD4 Count	<200	143	25.3	27.70	11.76	2.35	(1.52, 3.65)	1.69 × 10^−5^	****
	200–349	131	23.1	23.20	16.47	1.41	(0.96, 2.06)	0.078110547	
	350–499	103	18.2	16.89	16.47	1.03	(0.69, 1.52)	1	
	≥500	189	33.4	21.40	55.29	0.39	(0.31, 0.48)	2.53 × 10^−15^	****
	Missing	48	7.8	-	-	-	-	-	
Viral Load	50–999	9	1.5	2.03	0.00	-	-	-	
	1000–9999	50	8.5	11.26	0.00	-	-	-	
	≥10,000	529	89.7	80.86	100.00	0.81	(0.77, 0.84)	1.56 × 10^13^	****
	Missing	24	3.9	-	-	-	-	-	
Clade	Pure Subtype	267	43.5	53.38	17.65	3.02	(2.16, 4.23)	1.72 × 10^−16^	****
	Recombinant (CRF/URF)	214	34.9	41.89	16.47	2.54	(1.78, 3.63)	9.70 × 10^−10^	****
	Unclassified	2	0.3	0.45	0.00	-	-	-	
	Not Mentioned	131	21.3	4.28	65.88	0.06	(0.04, 0.10)	2.69 × 10^−58^	****
City	Jeddah	444	72.3	-	-	-	-	-	
	Dammam	170	27.7	-	-	-	-	-	

HIV-1 clade classification was based on genotypic data. “Pure Subtype” refers to viruses belonging to a single, well-defined subtype (e.g., A, B, C, D, F, G, H, J, K). “Recombinant (CRF/URF)” denotes circulating or unique recombinant forms that contain genetic segments from two or more subtypes (e.g., CRF02_AG, CRF01_AE, BC, BG). “Unclassified” represents isolates that could not be confidently assigned to either a pure subtype or a recombinant form based on available sequence information. “Not Mentioned” indicates records without clade information in the dataset. RR = relative risk; CI = confidence interval. Percentages (%) are calculated based on the total study population unless otherwise specified. Risk values represent the proportion within each category for Jeddah and Dammam, respectively. *p*-values are derived from comparative analyses between cities and are presented in scientific notation where applicable. Statistical significance is indicated as follows: * *p* < 0.05; ** *p* < 0.01; **** *p* < 0.0001. Cells marked with “-” indicate that estimates could not be calculated due to zero counts or insufficient data. “Missing” indicates unavailable data for the specified variable. Percentages were calculated using the total study population (*n* = 614).

**Table 2:** Prevalence of Drug Resistance Mutations by Drug Class.

Drug Class	Patients with ≥1 Mutation, *n* (%)	Total Unique Mutations, *n* (%)	Most Common Mutations (≥5%)
NRTI/NNRTI	511 (85.2%)	293 (99.7%)	R211K → 357 patients (59.5%) S68G → 57 patients (9.5%) D177E → 49 patients (8.2%) A98S → 42 patients (7.0%) Q207E → 40 patients (6.7%)
PI	571 (94.7%)	224 (99.6%)	M36I → 507 patients (84.1%) H69K → 476 patients (78.9%) L89M → 456 patients (75.6%) I13V → 391 patients (64.8%) K20I → 255 patients (42.3%)
INSTI	209 (36.4%)	74 (98.7%)	M50I → 44 patients (7.7%) L74I → 43 patients (7.5%) G163E → 30 patients (5.2%)

Percentages for patients are based on the total study population. “Most Common Mutations” include mutations observed in ≥5% of patients within each drug class. NRTI = nucleoside reverse transcriptase inhibitor; NNRTI = non-nucleoside reverse transcriptase inhibitor; PI = protease inhibitor; INSTI = integrase strand transfer inhibitor. Percentages of patients were calculated using the number of successfully genotyped sequences for each gene region: reverse transcriptase (RT), *n* = 600; protease (PI), *n* = 603; and integrase strand transfer inhibitor (INSTI), *n* = 574.

**Table 3:** Multi-Class Resistance Profiles of Patients across RT, PI, and INSTI Drug Classes.

Resistance Category	*n*	% of All Patients
RT + PI	328	53.40%
Triple-class (RT + PI + INSTI)	181	29.50%
PI only	41	6.70%
No resistance mutations	33	5.40%
PI + INSTI	21	3.40%
INSTI only	8	1.30%
RT only	2	0.30%

Resistance categories are defined based on the presence of ≥1 resistance-associated mutation within each drug class. Percentages are calculated using the total study population as the denominator. Percentages represent the proportion of the total study population (*n* = 614).

**Table 4:** Individual Mutations. (**A**) Frequency of Individual Mutations (RT, PI, INSTI). (**B**) Clinical Relevance of Major Mutations.

(**A**)				
**≥5% in This Cohort**				
**Mutation**	**Drug Class**	**Patients (*n*)**	**% Occurrence**	**Clinical Relevance**
R211K	RT	357	59.50%	Predominant RT polymorphism; no major resistance effect
S68G	RT	57	9.50%	Accessory mutation; may accompany NRTI-associated changes
D177E	RT	49	8.20%	Polymorphic; limited effect on drug susceptibility
A98S	RT	42	7.00%	Accessory NNRTI mutation; minor role unless combined with K103N or Y181C
Q207E	RT	40	6.70%	Background polymorphism; not linked to resistance
M36I	PI	507	84.10%	Common PI polymorphism; enhances resistance when combined with major PI mutations
H69K	PI	476	78.90%	Accessory PI mutation; prevalent natural variant
L89M	PI	456	75.60%	Polymorphism; may augment multi-PI resistance pathways
I13V	PI	391	64.80%	Polymorphic; minimal impact alone
K20I	PI	255	42.30%	Accessory PI mutation; contributes in combination with major PI mutations
M50I	INSTI	44	7.70%	Accessory INSTI mutation; minimal effect alone
L74I	INSTI	43	7.50%	Accessory mutation; may influence INSTI susceptibility when combined
G163E	INSTI	30	5.20%	Accessory mutation; supports major INSTI resistance pathways
K104R	RT	33	5.30%	Accessory NNRTI polymorphism
V60I	RT	31	5.10%	Background substitution
L10I	PI	31	5.00%	Accessory PI polymorphism
T74P	PI	30	5.00%	Background variant
(**B**)				
**<5% in This Cohort**				
**Mutation**	**Drug Class**	**Patients (*n*)**	**% Occurrence**	**Clinical Relevance**
M184V/I	RT (NRTI/NNRTI)	7	1.17%	High-level resistance to 3TC and FTC; reduces viral fitness; increases AZT/TDF susceptibility
K65R	RT (NRTI/NNRTI)	1	0.17%	Reduces susceptibility to TDF, ABC, ddI, 3TC; major NRTI resistance mutation
K103N	RT (NRTI/NNRTI)	5	0.83%	High-level NNRTI resistance (EFV, NVP); common global mutation
Y181C/I/V	RT (NRTI/NNRTI)	1	0.17%	Resistance to NNRTIs (NVP, EFV, RPV); reduces drug binding affinity
Q148H/K/R	INSTI	2	0.35%	Major INSTI resistance pathway; confers high-level resistance when combined with N155H
N155H	INSTI	1	0.17%	INSTI resistance mutation reducing RAL and EVG susceptibility

Panel (**A**) includes mutations observed in ≥5% of patients in this cohort. Panel (**B**) includes clinically relevant mutations observed in <5% of patients; these mutations were either present at low prevalence or not detected in this cohort but are included due to their well-established clinical significance in antiretroviral resistance according to the IAS–USA drug resistance list and Stanford HIVdb tool. Percentages are calculated based on the total study population. Mutations separated by slashes (e.g., Y181C/I/V) indicate alternative amino acid substitutions at the same position. EFV = efavirenz; NVP = nevirapine; RPV = rilpivirine; RAL = raltegravir; EVG = elvitegravir; 3TC = lamivudine; FTC = emtricitabine; AZT = zidovudine; TDF = tenofovir disoproxil fumarate; ABC = abacavir; ddI = didanosine. Percentages of patients were calculated using the number of successfully genotyped sequences for each gene region: reverse transcriptase (RT), *n* = 600; protease (PI), *n* = 603; and integrase strand transfer inhibitor (INSTI), *n* = 574.

**Table 5:** Co-occurrence of Frequent Mutations (≥5%).

Mutation Pair	Patients with Both (*n*)	% of Valid Patients
RT		
R211K + A98S	31	5.20%
R211K + Q207E	21	3.50%
R211K + D177E	19	3.20%
R211K + S68G	17	2.80%
A98S + D177E	2	0.30%
A98S + Q207E	2	0.30%
PI		
M36I + H69K	441	73.10%
M36I + L89M	421	69.80%
H69K + L89M	419	69.50%
M36I + I13V	367	60.90%
H69K + I13V	336	55.70%
L89M + I13V	334	55.40%
M36I + K20I	244	40.50%
I13V + K20I	239	39.60%
L89M + K20I	234	38.80%
H69K + K20I	221	36.70%
INSTI		
G163E + L74I	8	1.40%
G163E + M50I	2	0.35%
L74I + M50I	1	0.20%

Filtered single mutations that were ≥5% prevalence in the cohort (e.g., R211K, M36I, H69K, etc.). Percentages of patients were calculated using the number of successfully genotyped sequences for each gene region: reverse transcriptase (RT), *n* = 600; protease (PI), *n* = 603; and integrase strand transfer inhibitor (INSTI), *n* = 574.

## Data Availability

This study was based on archived clinical genotypic resistance reports. The underlying nucleotide sequence files were not available and therefore could not be deposited in GenBank. Anonymized clinical data supporting the findings of this study are available from the corresponding author upon reasonable request and subject to institutional ethical approval.

## Abbreviations

The following abbreviations are used in this manuscript:

HIV	Human Immunodeficiency Virus
HIV-1	Human Immunodeficiency Virus Type 1
TDR	Transmitted Drug Resistance
ART	Antiretroviral Therapy
RT	Reverse Transcriptase
PI	Protease Inhibitor
INSTI	Integrase Strand Transfer Inhibitor
NRTI	Nucleoside Reverse Transcriptase Inhibitor
NNRTI	Non-Nucleoside Reverse Transcriptase Inhibitor
DRM	Drug Resistance Mutation
SDRM	Surveillance Drug Resistance Mutation
WHO	World Health Organization
DHHS	Department of Health and Human Services
IAS–USA	International Antiviral Society–USA
BHIVA	British HIV Association
PCR	Polymerase Chain Reaction
RR	Relative Risk
CI	Confidence Interval
CRF	Circulating Recombinant Form
URF	Unique Recombinant Form
APOBEC	Apolipoprotein B mRNA Editing Catalytic Polypeptide-like
EFV	Efavirenz
NVP	Nevirapine
RPV	Rilpivirine
RAL	Raltegravir
EVG	Elvitegravir
3TC	Lamivudine
FTC	Emtricitabine
AZT	Zidovudine
TDF	Tenofovir Disoproxil Fumarate
ABC	Abacavir
ddI	Didanosine
CRF02_AG	Circulating Recombinant Form 02_AG
CRF01_AE	Circulating Recombinant Form 01_AE
CRF16_A2D	Circulating Recombinant Form 16_A2D
CRF43_02G	Circulating Recombinant Form 43_02G
CPX	Complex Recombinant Form
U	Unclassified subtype
